# Effectiveness of Catch-Up Vaccination Interventions Versus Standard or Usual Care Procedures in Increasing Adherence to Recommended Vaccinations Among Different Age Groups: Systematic Review and Meta-Analysis of Randomized Controlled Trials and Before-After Studies

**DOI:** 10.2196/52926

**Published:** 2024-07-23

**Authors:** Alessandra Fallucca, Walter Priano, Alessandro Carubia, Patrizia Ferro, Vincenzo Pisciotta, Alessandra Casuccio, Vincenzo Restivo

**Affiliations:** 1 Department of Health Promotion, Mother and Child Care, Internal Medicine and Medical Specialities University of Palermo Palermo Italy; 2 School of Medicine University Kore of Enna Enna Italy

**Keywords:** vaccine strategies, catch-up interventions, recall intervention, vaccination coverage, multicomponent, education, remind, reward, vaccination, vaccine, adherence, systematic review, meta-analysis, immunization, health care based, multidimensional intervention, education based, vaccine literacy, PRISMA

## Abstract

**Background:**

To address the global challenge of vaccine hesitancy, the Strategic Advisory Group of Experts on Immunization strongly promotes vaccination reminder and recall interventions. Coupled with the new opportunities presented by scientific advancements, these measures are crucial for successfully immunizing target population groups.

**Objective:**

This systematic review and meta-analysis aims to assess the effectiveness of various interventions in increasing vaccination coverage compared with standard or usual care. The review will cover all vaccinations recommended for different age groups.

**Methods:**

In February 2022, 2 databases were consulted, retrieving 1850 studies. Following the PRISMA (Preferred Reporting Items for Systematic Reviews and Meta-Analyses) guidelines, 79 manuscripts were included after the assessment phase. These comprised 46 trials/randomized controlled trials (RCTs) and 33 before-after studies. A meta-analysis using a random-effects model was performed with STATA software (version 14.1.2). The selected outcome was the risk ratio (RR) of vaccination coverage improvement effectiveness. Additionally, meta-regression analyses were conducted for the included manuscripts.

**Results:**

The analyses showed an overall efficacy of RR 1.22 (95% CI 1.19-1.26) for RCTs and RR 1.70 (95% CI 1.54-1.87) for before-after studies when considering all interventions cumulatively. Subgroup analyses identified multicomponent interventions (RR 1.58, 95% CI 1.36-1.85) and recall clinical interventions (RR 1.24, 95% CI 1.17-1.32) as the most effective in increasing vaccination coverage for RCTs. By contrast, educational interventions (RR 2.13, 95% CI 1.60-2.83) and multicomponent interventions (RR 1.61, 95% CI 1.43-1.82) achieved the highest increases for before-after studies. Meta-regression analyses indicated that the middle-aged adult population was associated with a higher increase in vaccination coverage (RCT: coefficient 0.54, 95% CI 0.12-0.95; before-after: coefficient 1.27, 95% CI 0.70-1.84).

**Conclusions:**

Community, family, and health care–based multidimensional interventions, as well as education-based catch-up strategies, effectively improve vaccination coverage. Therefore, their systematic implementation is highly relevant for targeting undervaccinated population groups. This approach aligns with national vaccination schedules and aims to eliminate or eradicate vaccine-preventable diseases.

## Introduction

The immunization programs are specifically designed to maximize the health benefits for the population by offering the most appropriate vaccinations for different age groups and types of patients [1]. The effectiveness of vaccination programs is based on a high uptake level. In addition to providing direct protection for vaccinated individuals, these programs offer indirect protection to the community by decreasing the risk of infection [2].

Although vaccination is one of the most successful public health interventions, global immunization coverage rates remain unsatisfactory. In 2021, nearly 25 million children under the age of 1 year missed their routine diphtheria-tetanus-pertussis vaccinations. Additionally, human papillomavirus (HPV) vaccination coverage among girls in the least developed countries was only 15% [3]. Undervaccination can be attributed to a lack of health services available to the population, lower availability of vaccines for mass immunization programs, and difficulties in accessing these services in terms of both space and time [4]. Insufficient budgets are one of the main barriers preventing health governments from providing access to mass vaccination in low-income countries [5].

Although the health governments of the most developed countries are strongly implementing national immunization programs, introducing new vaccines, and expanding vaccination offers, coverage rates are still far from desirable targets. This shortfall has resulted in outbreaks of vaccine-preventable diseases, leading to hospitalizations and, in some cases, death [6,7]. The literature examining the acceptance of routine vaccinations for adolescents (such as the HPV vaccine) and vaccines recommended for older adults or those with chronic and disabling conditions (such as influenza, pneumococcal, and herpes zoster vaccines) indicates critical issues with uptake [8,9].

Vaccine hesitancy, defined as “the reluctance or refusal to vaccinate despite vaccine availability,” has gained recognition as a top threat to global health because it could undermine successful and cost-effective vaccination programs worldwide [10]. The main factors contributing to vaccine hesitancy are a lack of awareness about the benefits of vaccination, concerns regarding short- or long-term side effects of vaccines, general distrust in immunization practices, and doubts about the high number of vaccines administered according to schedules [11]. Furthermore, the growing complexity of vaccination programs, with the introduction of new vaccines and the high number of recommended booster doses, could represent an obstacle to achieving optimal coverage. This complexity can cause difficulties in adherence and delays in vaccinating the target population [12].

The decline in vaccinations threatens to strain health systems with outbreaks of vaccine-preventable diseases. Several attempts have been made to identify approaches that increase immunization coverage, such as vaccine information campaigns, promotional and educational messages for patients and health care professionals, and the use of reminders and various mobile apps [12,13]. Active vaccine catch-up interventions can be an extremely useful tool for improving adherence to vaccination practices. Experience and research can help identify the most effective vaccination strategies.

This systematic review and meta-analysis aims to evaluate vaccine adherence across various catch-up methods targeting different age groups. Additionally, we aimed to identify the most effective vaccination recall strategies compared with standard or usual care procedures, based on randomized controlled trials (RCTs) and before-after studies.

## Methods

### Study Guidelines

For this systematic review, we followed the PRISMA (Preferred Reporting Items for Systematic Reviews and Meta-Analysis; Multimedia Appendix 1) statement guidelines [14] to ensure transparency and thorough reporting of both the review process and results. The review protocol was registered on PROSPERO under the registration number CRD42022307311 and it can be accessed online [15].

### Search Strategy and Selection Criteria

Two literature databases, PubMed/MEDLINE and Scopus, were utilized for this review. The literature search commenced on February 14, 2022, using a combination of free-text words and Medical Subject Headings (MeSH). The search strategy incorporated general terms such as “vaccine,” “effectiveness,” and “improvement,” along with specific terms related to catch-up intervention implementation. The search strings obtained are detailed in Multimedia Appendix 2.

The Population, Intervention, Comparison, Outcomes and Study (PICOS) criteria were applied to select studies, encompassing populations of all ages without restrictions on country or length of follow-up. Eligible participants were those eligible for vaccination and receiving a catch-/mop-/keep-up intervention involving reminders or recalls. The objective was to evaluate the intervention-dependent vaccination coverage improvement effectiveness (VCIE), which is a composite outcome created by assessing both the improvement in vaccination coverage and the completion of vaccination series, in comparison to standard vaccination practices. During both the screening and assessment phases, authors excluded research articles based on the following criteria: if the topic or outcome did not align with the review’s objective, if the study did not use an RCT or before-after study design, if the studies lacked vaccination coverage data, or if there were insufficient data regarding the “before” or “control” group or description of catch-up/recall intervention. Furthermore, non-English manuscripts and articles whose full texts were unavailable were excluded.

During the screening phase, a total of 6 reviewers applied the inclusion criteria (AF, WP, AC, PF, VP, and VR). This process involved 3 pairs of independent reviewers, each consisting of 2 reviewers. These pairs evaluated the title and abstract of each identified article. Subsequently, the same pairs performed the assessment phase by evaluating the full text of the selected articles. In case of disagreements or doubts, a formal reconciliation process was undertaken to reach a consensus among the reviewers. If needed, the intervention of another reviewer was sought to resolve the issue and make a final decision.

### Data Analysis

For studies that met the inclusion criteria, a full-text review and data extraction were conducted using a standardized template. This template included outcome measures and demographics of the study population, such as study design, country, study recruitment range, follow-up time, primary objective, outcome, intervention type, number of patients enrolled, gender distribution, age range, and gross national income (GNI). The variable “follow-up time since intervention” was categorized as follows: 0 to <6 months (short), 6 to <12 months (medium), and more than 12 months (long).

Furthermore, given the study’s objective to detail the effectiveness of various types of catch-up reminder or recall vaccination interventions on coverage rates, the variable “intervention type” was further classified based on an existing reference [16]. The intervention categories were delineated as follows: “remind” studies were divided into clinical, messaging, web, active calls, and object; “reward” studies; and “educational” studies. In cases where multiple types of vaccination interventions were combined and administered, they were classified under the category of “multicomponent” interventions.

According to the Grading of Recommendations Assessment, Development, and Evaluation (GRADE) classification [17], bodies of evidence from RCTs are a priori regarded as “high”-quality evidence, whereas evidence from observational studies starts as “low”-quality evidence. To further define and assess the risk of bias in each included study, 2 quality assessment score tools specific to the study designs were utilized. For RCTs, the assessment tool shown in Table 2 [18] was used, and for before-after studies, the tool in Table 5 [19] was used. The Duval and Tweedie nonparametric trim-and-fill method was used to adjust for the effect of publication bias. This method is utilized to account for hypothetical small missing null or negative studies, thereby providing a more balanced assessment of the data.

Study-level data were recorded in Excel spreadsheets (Microsoft Excel 2010). Risk ratios (RRs) and corresponding 95% CIs of VCIE were directly calculated to evaluate the effect of different intervention types on the receipt of immunizations. Separate analyses were conducted for RCTs and before-after studies. If data on the main outcome (VCIE) were available from more than 1 study, a random effects model meta-analysis was used to pool the data. The results were stratified based on the “intervention category” variable, and pooled RRs and risk differences were computed for each intervention category. These analyses were conducted using STATA software (version 14.2.1; StataCorp). Between-study variation was estimated by comparing each study’s result with a Mantel-Haenszel fixed-effect meta-analysis. The extent of heterogeneity was quantified using *I*^2^. Testing for publication bias was conducted separately for RCTs and before-after studies for the main outcome. Additionally, meta-regression analyses were performed using the following summary measures: an estimate of between-study variance (tau), the proportion of between-study variance (adjusted *R*^2^), the percentage of residual variation due to heterogeneity (*I*^2^), and a joint test for all covariates (model F) with Knapp-Hartung modification (prob>*F*).

## Results

### Overview

**Figure 1:**
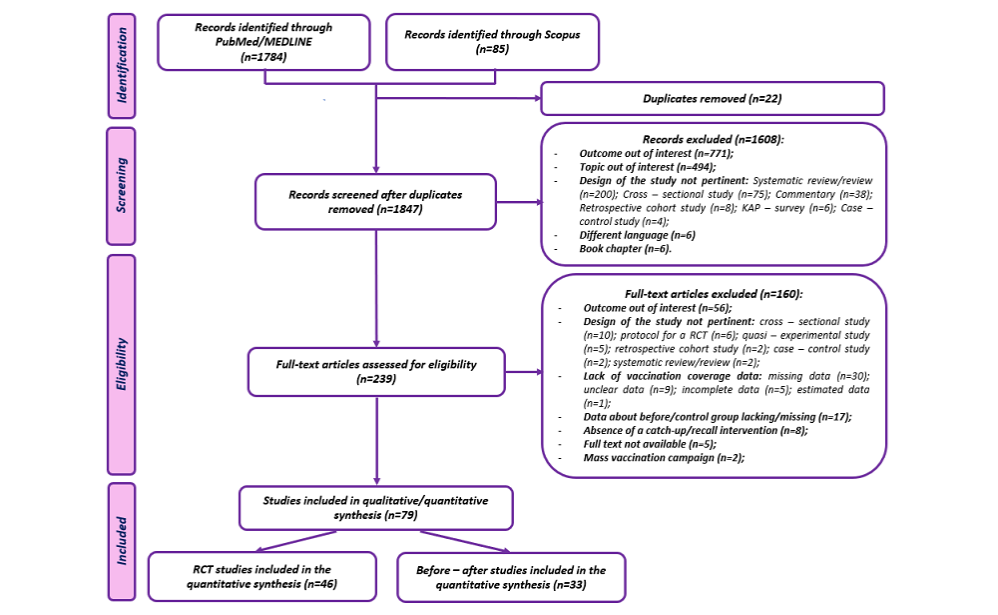
PRISMA (Preferred Reporting Items for Systematic Reviews and Meta-Analysis) flow diagram of studies selection.

A total of 1869 research articles were identified from the literature databases, with 1784 (94.45%) retrieved from the PubMed/MEDLINE platform and 85 (4.55%) from Scopus. After removing duplicates (n=22), 1847 records underwent screening based on titles and abstracts. Among these, 239 full-text articles were assessed for eligibility, and ultimately, 79 studies were included in the data extraction and qualitative synthesis. Specifically, 46 (58%) of these studies were RCTs, and 33 (42%) were before-after studies included in the meta-analysis. The main reasons for excluding studies were as follows: outcomes not aligned with the review’s interest (n=56) and studies not utilizing an RCT or before-after study design (n=29). A summary of the screening process and exclusions is depicted in the PRISMA flow diagram (Figure 1).

### Characteristics, Quality Score, and Results of Meta-Analysis and Meta-Regression for RCT Studies

Of the included RCTs, 20 studies (43%) were conducted in the American continent, with 18 of them (39%) in the United States. Additionally, 11 studies (24%) were conducted in Asia and 9 studies (20%) in Europe. Only 5 studies (11%) were conducted in Africa and 1 study was conducted in Australia. Regarding the age groups targeted by the interventions, the studies were most frequently focused on the “infant-preschool” category in 46% of cases (n=21), followed closely by the “child-adolescent” category in 43% of cases (n=20). The “middle-aged adult” category was addressed in 15% of studies (n=7). According to the classification of intervention types, “multicomponent” studies were identified most frequently, accounting for 48% (n=22) of the total. Following this, “educational” studies comprised 24% (n=11) of the included studies. The “reward” category was the least represented, with only 4% (n=2) of the studies falling under this category. Among the reminder interventions, the “clinical” and “messaging” subcategories were the most populated, accounting for 22% (n=10) and 28% (n=13) of the included studies, respectively (Table 1).

The quality score evaluation of the included RCT studies revealed that 2 studies [20,21] received the maximum score of 5. Several studies were assigned a score of 1 point, including 8 “multicomponent” studies [22-29], 5 “educational” studies [22,30-33], 3 “reminder” studies [34-36], and 2 “reward” studies [37,38]. Only 2 studies [39,40] received the minimum score of 0. Adequacy of randomization was found in 27 studies (59%), while blinding was reported in only 2 studies (4%) out of the 46 included studies (Table 2).

The overall results of meta-analyses for all RCT studies demonstrated an RR of 1.22 (*P*<.001), indicating an increase in VCIE across all types of interventions included in the RCT sample. Further details on heterogeneity and significance tests for all intervention categories are provided in Multimedia Appendix 3. The most effective interventions are extensively detailed in Multimedia Appendix 4, which includes the forest plot of RCTs. The highest efficacy was reported for “multicomponent” interventions, with a risk ratio (RR) of 1.58 (*P*<.001) (see Figure S1A in Multimedia Appendix 4), followed by “reminder clinical” studies, which exhibited an RR of 1.24 (*P*<.001; see Figure S1B in Multimedia Appendix 4). Furthermore, “educational” interventions (RR 1.15; *P*<.001) and “reminder messaging” interventions (RR 1.14; *P*<.001) demonstrated a positive effect on VCIE. Forest plots summarizing results for other types of recall interventions can be found in Figure S1C, D in Multimedia Appendix 4.

For RCT studies, meta-regression analyses were conducted to obtain the best fitting model, including the following variables: publication year, continent, GNI, age category, and follow-up time since intervention. According to this analysis, interventions conducted in the European continent (coefficient 1.001; *P*=.03) and targeting the “adult-middle age” population (coefficient 0.537; *P*=.012) were the most effective in increasing vaccination coverage (Table 3).

**Table 1:** Characteristics of RCT included studies.^a^

Publication	Country	Study year range	Follow-up time since intervention	Main outcome (vaccination rate/coverage)	Intervention type	Mean age among the intervention and control groups	Number of patients enrolled	Quality score assigned
Rodewald et al [20]	United States	1994-1995	18 months	Full series completion for all vaccines	Education (outreach educational campaign)	0-12 months	2741	5
LeBaron et al [41]	United States	1996-1998	36 months	dTPa^b^, polio, MMR^c^, and Hib^d^ series completion	Education (in-person/telephone call)	1-14 months	3050	3
Kimura et al [22]	United States	May-October 2002	3 months	Influenza	Education (educational campaign for health care workers)	18-65 years	2338	1
Gilkey et al [30]	United States	2011	12 months	dTPa and meningococcal	Education (in-person and webinar-delivered AFIX^e^ educational sessions)	11-12 years (n=32,676); 13-18 years (n=74,767)	107,443	1
Brewer et al [42]	United States	2015	6 months	HPV^f^ 9 full series completion	Education (informative announcements vs face-to-face conversation)	11-12 years	17,173	3
Wong et al [43]	China	2013-2015	0.5 months	Influenza (adherence to self-reported vaccination)	Education (face-to-face short individual educational session for pregnant women)	33-34 years	321	3
Brown et al [31]	Nigeria	2012-2013	12 months	All vaccines	Education (nurse-led educational sessions in primary health care centers)	0-12 weeks (intervention on parents)	300	1
Hu et al [44]	China	2014	12 months (from birth to first year of life)	Full series completion of all vaccines	Education (educational sessions for pregnant women)	Adults (women aged 20-30 years); infants up to the first year of life	1252	3
Esposito et al [45]	Italy	2015-2016	8 months	dTPa and meningitis ACWY	Education (multiple web-based educational programs)	Adolescents	615	2
Lemaitre et al [32]	Canada	2014	24 months	Full series completion of all vaccines	Education (motivational interview-based educational strategy)	Adults (mothers); children at 2 years of age	2717	1
Muñoz-Miralles et al [33]	Spain	October 2017 to March 2018	6 months	Influenza	Education (face-to-face educational intervention)	Middle aged to aged >80 years: ≥60 years healthy; ≥60 years with risk factors; <60 years with risk factors; others	524	1
Rodewald et al [20]	United States	1994-1995	18 months	Full series completion of all vaccines	Multicomponent (combined: tracking + outreach with prompting)	0-12 months	2741	5
LeBaron et al [41]	United States	1996-1998	36 months	dTPa, polio, MMR, and Hib full series completion	Multicomponent (autodialer with outreach backup)	1-14 months	3050	3
Szilagyi et al [21]	United States	1998-2000	18 months	dTPa and HBV^g^	Multicomponent (audiotaped telephone reminders and active calls)	11-14 years	3006	5
Kimura et al [22]	United States	May to October 2002	3 months	Influenza	Multicomponent (educational campaign and vaccination day for health care workers)	18-65 years	2271	1
Schwarz et al [23]	United States	1995	3 months	HBV	Multicomponent (video on HBV, gift packages for children, and cash gifts for caregivers)	2-18 years	328	1
Humiston et al [46]	United States	2002-2004	0 months	Influenza	Multicomponent (patient tracking, recall, outreach, and provider prompts)	<65 years	3752	3
Mantzari et al [47]	United Kingdom	February 2010 to March 2010	6 months	HPV series initiation and completion	Multicomponent (first-time invitees: letter, voucher [financial incentive], and SMS text messages vs previous nonattenders: letter, voucher [financial incentive], SMS text messages)	16-18 years	1000	2
Chamberlain et al [24]	United States	2012-2013	3 months after giving birth	dTPa and influenza	Multicomponent (multilevel intervention involving clinic, provider, and patient)	26.9-27.5 years (perinatal vaccination)	325	1
Richman et al [25]	United States	August 2011 to December 2013	7 months	HPV full series completion	Multicomponent (SMS text messages + emails)	18-26 years	283	1
Zimmerman et al [48]	United States	2014-2015	9 months	HPV 9 full series completion	Multicomponent (multimodal intervention: facilitations for access to vaccination services, communications with patients, SMS text messages, calls, and training sessions)	11-17 years	10,861	3
Brown et al [31]	Nigeria	2012-2013	12 months	All vaccines completion	Multicomponent (reminder intervention + providers training)	0-3 months (intervention on parents)	297	1
Ma et al [49]	United States	Not mentioned	12 months	HBV	Multicomponent (training of providers and involvement of church through messaging)	≥18 (mean age 51.6) years	2212	2
Nagar et al [50]	India	August to December 2015	6 months	dTPa full series completion (within 180 days from birth)	Multicomponent (necklace with a pendant that records immunity data and provides voice reminders)	0-3 months	137	2
Esposito et al [45]	Italy	2015-2016	8 months	dTPa, meningitis ACWY, and meningitis B	Multicomponent (educational program on the website + face-to-face lessons)	11.6-16.4 years	636	2
Wallace et al [51]	Indonesia	January 2016 to July 2016	7 months	dTPa full series completion (third dose)	Multicomponent (home-based records + sticker)	0-12 months	3616	3
Borgey et al [52]	France	November 2014 to March 2015	0 months	Influenza	Multicomponent (multilevel intervention approach)	18-65 years (health care professionals)	4069	3
Currat et al [26]	Switzerland	April 2016 to October 2016	5 months	Influenza	Multicomponent (preemployment health test check: face-to-face intervention + reminder: information leaflet)	31-33 years	379	1
Menzies et al [27]	Australia	February 2015 to December 2015	36 months	Administering all vaccines in a timely manner	Multicomponent (SMS text messages through the VaxSMS app, calendar reminder)	2-8 months (intervention on parents)	1594	1
Liao et al [28]	China	October 2017 to December 2017	5 months	Influenza	Multicomponent (vaccination reminders + pressure component, WhatsApp discussion group)	6 months to 6 years	365	1
Yunusa et al [29]	Nigeria	November 2019	6 months	dTPa, HBV, and Hib full series completion (third dose)	Multicomponent (SMS text messages and calls)	20.2-33 years	554	1
Levine et al [53]	Ghana	March 2019 to April 2019	3 months	Administering all vaccines in a timely manner	Multicomponent (mobile phone–based reminders + incentives to health workers and caregivers)	28.5-29.8 years (mothers interviewed); outcome for neonatal vaccination	467	3
Kagucia et al [54]	Kenya	December 2016 to March 2017	6 months	MMR 1 timeliness vaccination	Multicomponent (SMS text messages and financial incentive)	6-8 months	537	3
Brown et al [34]	Nigeria	2012-2013	13 months	dTPa full series completion	Remind active call	3 weeks	614	1
Levine et al [53]	Ghana	March 2019 to April 2019	3 months	Administering all vaccines in a timely manner	Remind active call (phone call with health care worker reminder)	28.5-29.8 years (mothers interviewed); outcome for neonatal vaccination	479	3
Kimura et al [22]	United States	May to October 2002	3 months	Influenza	Clinical reminder (vaccination day for health care workers)	18-65 years (health care workers)	2349	1
Fiks et al [39]	United States	September 2004 to August 2005	Check at 2 years of age	All vaccines captured immunization	Clinical reminder (electronic health record–based clinical reminder)	0-2 years	3217	0
Andersson et al [55]	Pakistan	2005-2007	24 months	MMR and dTPa full series completion	Clinical reminder (informed discussion about vaccination)	12-23 months (intervention on parents)	904	3
Gilkey et al [30]	United States	2011	12 months	dTPa and meningococcal	Clinical reminder (in-person consultations)	11-12 years (n=32,676); 13-18 years (n=74,767)	69,051	1
Yoo et al [40]	United States	2009-2011	12 months	Influenza	Clinical reminder (school-located vaccination against flu in 2009-2010 and 2010-2011)	6 months to 18 years	13,561	0
Brown et al [31]	Nigeria	2012-2013	12 months	Full series completion for all vaccines	Clinical reminder	0-3 months	298	1
Kriss et al [56]	United States	2013	2 months after giving birth	dTPa	Clinical reminder (messaging iBook)	25.4-27.5 years (women in the perinatal period)	73	3
Hu et al [35]	China	2014	24 months	Varicella	Clinical reminder (messaging iBook)	25-26 years (parents); outcome for children at 2 years of age	136	1
Wallace et al [51]	Indonesia	January 2016 to July 2016	7 months	dTPa full series completion	Clinical reminder (home-based records)	0-12 months	3616	3
Blanchi et al [57]	France	May 2018 to May 2019		dTPa-inactivated polio vaccine	Clinical reminder (catch-up strategy during hospitalization)	65-97 years (hospitalized patients)	162	3
Rodewald et al [20]	United States	1994-1995	18 months	Full series completion for all vaccines	Remind messaging (prompting)	0-12 months	2741	5
Quinley and Shih [58]	United States	1999-2000	3 months	Pneumococcal (African American vs American)	Remind messaging (telephone call reminder)	<65 years	218 (African American); 732 (American)	3
LeBaron et al [41]	United States	1996-1998	36 months	dTPa, polio, MMR, and Hib full series completion	Remind messaging (autodialer: automated telephone or email reminders)	1-14 months	3050	3
Irigoyen et al [59]	United States	2001 (July to December)	6 months	dTPa	Remind messaging (continuous messaging reminders)	6 weeks to 15 months (outcome at 6 months after the intervention)	1662	3
Muehleisen et al [60]	Switzerland	2003	9 months	All vaccines completion	Remind messaging (written letter reminders)	2 months to 17 years; intervention on parents (postdischarge catch-up immunization)	532	3
Rand et al [61]	United States	2013-2014	9 months (July 2013 to March 2014)	HPV 9 full series completion	Remind messaging (reminder SMS text messages)	11-16 years	3812	2
Chen et al [62]	China	2013-2015	14 months (December 2013 to January 2015)	BCG^h^, HBV, dTPa-inactivated polio vaccine, MMR full series completion	Remind messaging (smartphone app: reminder vaccination SMS text messages)	0-13 months (children); intervention on parents	214	3
Domek et al [63]	Guatemala	2013	6 months	All vaccines (pentavalent, rotavirus, polio, pneumococcal) series completion	Remind messaging (SMS text message reminders)	2-4 months (infants); intervention on parents	321	3
Hu et al [35]	China	2014	24 months	Varicella	Remind messaging (video messaging)	25-26 years (parents); outcome for children at 2 years of age	136	1
Menzies et al [27]	Australia	February 2015 to December 2015	36 months	All vaccines timeliness vaccination	Remind messaging (SMS text message reminders through the VaxSMS app)	2-8 months (mean age 4 months); intervention on parents	1594	1
Liao et al [28]	China	October 2017 to December 2017	5 months	Influenza	Remind messaging (vaccination reminders through WhatsApp)	6 months to 6 years	365	1
Qin et al [36]	China	2019-2020	10 months	Varicella	Remind messaging (telephone notification vs written notification)	Telephone notification: 2.1-12.2 years (mean age 4.0 years); written notification: 2.1-7.3 years (mean age 3.8 years)	800	1
Kagucia et al [54]	Kenya	December 2016 to March 2017	6 months	MMR vaccine 1 timeliness vaccination	Remind messaging (SMS text messages)	6-8 months	537	3
Nagar et al [50]	India	August to December 2015	6 months	dTPa full series completion (third dose)	Remind object (pendant recording the vaccination history of the child)	0-6 months (outcome assessed within 180 days from birth)	123	2
Siddiqi et al [64]	Pakistan	July 2017 to October 2017	Until the administration of the measles-1 vaccine or until 12 months of age	dTPa, HBV, Hib full series completion (third dose); MMR vaccine 1	Remind object (Alma Sana Bracelet vs star bracelet)	Infants: 0.2-5 weeks (mean age 2.5 years); mothers: 20.8-31.4 years (mean age 26.6 years); fathers: 26.1-37.7 years (mean age 31.8 years)	2497	3
Irigoyen et al [59]	United States	July to December 2001	6 months	dTPa vaccination rate at 6 months after intervention in infants	Remind web	6 weeks to 15 months	1662	3
Kriss et al [56]	United States	2013	2 months after giving birth	dTPa (prenatal period)	Remind web (messaging video)	25.3-25.8 years	73	3
Menzies et al [27]	Australia	February 2015 to December 2015	36 months	All vaccines timeliness vaccination	Remind web (email calendar reminders)	2-8 months (mean age 4 months)	1594	1
Sääksvuori et al [65]	Finland	June 2018 to October 2018	5 months	Influenza, all vaccines (low coverage in Western region)	Remind web (email: individual benefits reminder vs individual and social benefit reminder)	>65 years (mean age 75.5 years)	7398	3
Chandir et al [37]	Pakistan	2006-2007	16 months	dTPa full series completion	Reward (food/medicine coupon incentives)	0-6 months	3059	1
Alessandrini et al [38]	France	October 2016 to January 2017	4 months	Influenza	Reward (free vaccination at prenatal consultation ward)	27.1-38.2 years	248	1

^a^Variables reported were author’s first name, publication year, country, recruitment study year range, follow-up period since the intervention, outcome(s), intervention type and category, mean age range among controls/interventions, and number of enrolled patients.

^b^dTPa: diphtheria, tetanus, and acellular pertussis.

^c^MMR: measles, mumps, and rubella.

^d^Hib: *Hemophilus influenzae* type b.

^e^AFIX: assessment, feedback, incentives, exchange.

^f^HPV: human papillomavirus.

^g^HBV: hepatitis B virus.

^h^BCG: bacillus Calmette-Guérin.

**Table 2:** The scoring system^a^ used for randomized controlled trials.

Study	Randomization				Blinding				An account of all patients		
	Mentioned	Appropriate	Inappropriate or not mentioned	Mentioned		Appropriate	Inappropriate or not mentioned	Fate of all patients known		Total score	
Quinley and Shih [58]	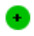	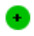	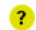	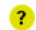		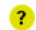	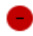	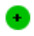		3	
LeBaron et al [41]	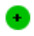	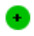	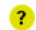	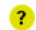		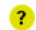	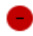	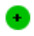		3	
Irigoyen et al [59]	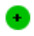	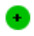	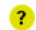	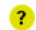		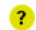	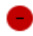	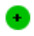		3	
Szilagyi et al [21]	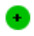	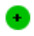	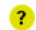	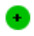		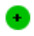	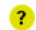	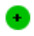		5	
Fiks et al [39]	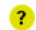	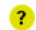	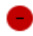	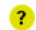		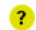	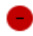	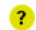		0	
Muehleisen et al [60]	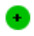	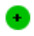	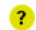	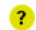		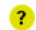	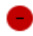	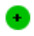		3	
Kimura et al [22]	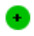	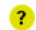	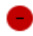	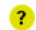		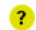	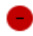	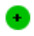		1	
Schwarz et al [23]	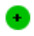	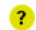	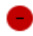	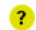		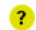	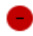	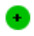		1	
Andersson et al [55]	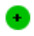	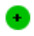	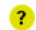	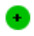		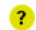	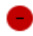	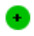		3	
Chandir et al [37]	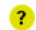	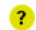	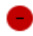	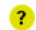		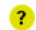	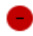	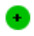		1	
Humiston et al [46]	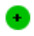	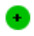	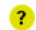	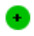		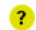	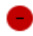	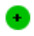		3	
Rand et al [61]	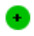	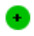	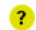	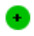		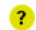	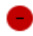	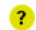		2	
Gilkey et al [30]	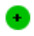	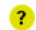	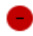	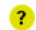		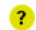	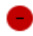	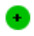		1	
Mantzari et al [47]	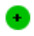	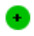	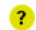	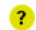		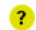	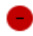	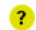		2	
Yoo et al [40]	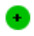	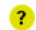	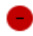	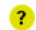		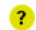	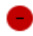	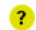		0	
Chamberlain et al [24]	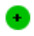	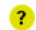	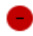	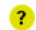		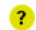	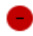	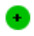		1	
Richman et al [25]	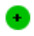	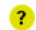	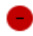	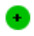		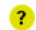	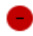	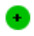		1	
Brewer et al [42]	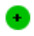	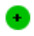	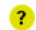	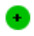		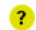	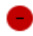	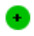		3	
Zimmerman et al [48]	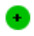	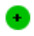	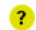	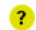		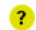	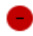	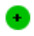		3	
Wong et al [43]	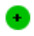	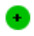	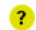	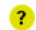		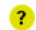	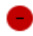	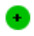		3	
Chen et al [62]	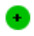	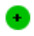	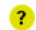	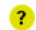		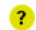	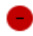	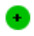		3	
Domek et al [63]	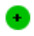	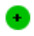	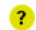	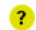		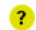	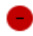	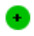		3	
Brown et al [31]	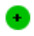	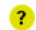	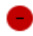	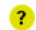		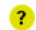	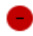	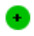		1	
Brown et al [34]	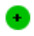	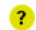	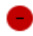	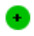		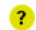	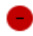	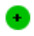		1	
Ma et al [49]	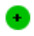	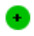	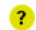	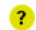		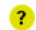	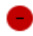	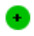		2	
Hu et al [44]	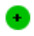	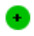	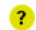	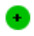		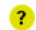	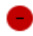	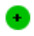		3	
Kriss et al [56]	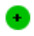	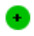	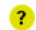	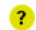		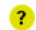	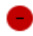	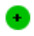		3	
Hu et al [35]	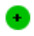	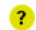	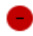	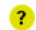		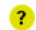	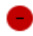	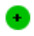		1	
Nagar et al [50]	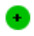	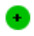	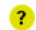	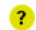		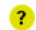	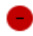	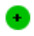		2	
Esposito et al [45]	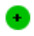	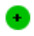	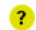	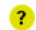		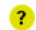	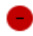	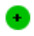		2	
Rodewald et al [20]	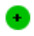	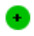	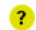	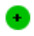		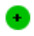	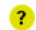	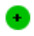		5	
Wallace et al [51]	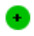	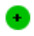	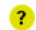	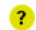		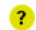	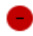	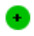		3	
Alessandrini et al [38]	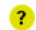	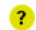	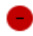	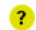		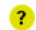	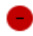	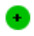		1	
Borgey et al [52]	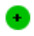	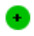	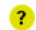	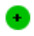		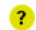	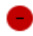	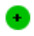		3	
Lemaitre et al [32]	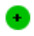	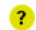	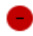	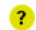		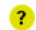	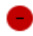	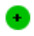		1	
Currat et al [26]	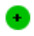	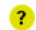	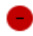	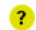		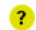	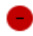	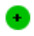		1	
Siddiqi et al [64]	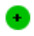	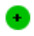	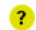	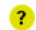		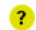	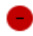	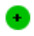		3	
Blanchi et al [57]	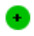	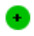	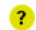	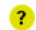		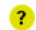	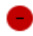	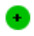		3	
Menzies et al [27]	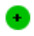	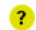	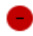	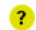		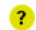	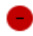	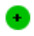		1	
Liao et al [28]	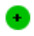	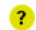	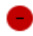	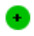		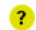	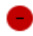	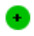		1	
Yunusa et al [29]	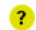	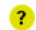	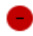	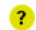		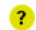	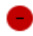	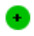		1	
Qin et al [36]	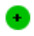	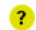	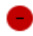	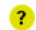		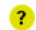	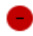	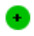		1	
Levine et al [53]	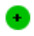	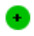	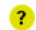	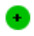		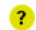	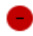	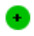		3	
Muñoz-Miralles et al [33]	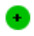	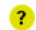	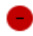	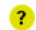		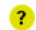	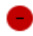	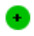		1	
Kagucia et al [54]	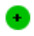	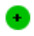	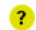	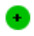		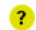	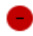	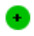		3	
Sääksvuori et al [65]	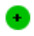	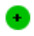	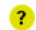	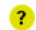		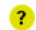	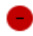	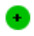		3	

^a^1 additional point for the appropriate item, while 0 points are awarded if not appropriate; –1 point is awarded for not mentioned or not the appropriate method. The minimum score for each analyzed section is 0.

**Table 3:** Meta-regression of randomized controlled trials.

Variable			Coefficient		SE		*t* test (*df*)		*P* value		95% CI
Publication year			0.001		0.017		0.05 (84)		.96		–0.034 to 0.036
**Continent**											
	Africa	0.449		0.594		0.76 (84)		.45		–0.736 to 1.635	
	America	0.084		0.426		0.20 (84)		.84		–0.766 to 0.935	
	Europa	1.003		0.448		2.24 (84)		*.*03^a^		0.108 to 1.898	
	Asia	0.192		0.485		0.40 (84)		.69		–0.775 to 1.158	
	Gross national income	0.013		0.163		0.08 (84)		.94		–0.312 to 0.338	
**Age**											
	Infant-preschool	0.069		0.209		0.33 (84)		.74		–0.349 to 0.487	
	Children-adolescent	0.280		0.182		1.54 (84)		.13		–0.082 to 0.643	
	Adult-middle age	0.537		0.208		2.57 (84)		*.*01^a^		0.121 to 0.954	
	Aged	–0.075		0.234		–0.32 (84)		.75		–0.542 to 0.392	
**Follow-up (months)**											
	6	–0.238		0.669		–0.36 (84)		.73		–1.573 to 1.097	
	12	0.277		0.685		0.40 (84)		.69		–1.089 to 1.644	
	>12	0.173		0.694		0.25 (84)		.80		–1.210 to 1.558	

^a^Statistically significant results.

### Characteristics, Quality Score, and Results of Meta-Analysis and Meta-Regression for Before-After Studies

Among the 33 before-after studies included, 42% (n=14) were conducted in the American continent, predominantly in the United States (n=13, 39%). Europe accounted for 30% (n=10) of the studies, followed by Asia with 15% (n=5) of the studies. Additionally, 9% (n=3) of the studies were carried out in Africa (Egypt, Kenya, and Nigeria), while only 3% (n=1) were conducted in Australia.

The most prevalent “age categories” were the “child-adolescent” category, accounting for 58% (n=19), followed by the “adult-middle age” category, which comprised 39% (n=13) of the studies.

The most frequent “intervention category” was “multicomponent” (n=16), followed by “educational” studies (n=9). Within the “reminder” studies, 4 were classified as “clinical,” 2 as “messaging,” and 1 each as “active call” and “reward” studies. Further information is provided in Table 4.

The scoring system used to assess bias in before-after studies examined various items, with 1 additional point assigned each time the item in question was present. The items in question were clearly stated study question or objective (32/33 studies, 97%); prespecified and clearly described eligibility/selection criteria for the study population (28/33, 85%); study participants representative of those who would be eligible for the test/service/intervention in the general/clinical population of interest (28/33, 85%); whether all eligible participants who met prespecified entry criteria were enrolled (15/33, 45%); sample size large enough to provide confidence (18/33, 55%); test/service/intervention clearly described and delivered consistently across study population (17/33, 52%); prespecified, clearly defined, valid, reliable, and consistently assessed outcome measures across all study participants (19/33, 58%); people who assessed outcomes blinded to participant exposures/interventions (0/33, 0%); losses to follow-up after baseline 20% or less and whether those lost to follow-up were considered in the analysis (19/33, 58%); presence of changes in outcome measures from pre- to postintervention with *P* values statistically examined for pre-post changes (27/33, 82%); and outcome measures taken several times before the intervention/after the intervention (15/33, 45%). The evaluation of the quality score of the before-after studies revealed that 8 studies attained the maximum score of 8. Among these, 4 were categorized as “educational” [66-69], 2 as “multicomponent” [70,71], and 2 as “reminder clinical” [72,73]. By contrast, the lowest score of 4 was assigned to 2 studies: 1 categorized as “reminder clinical” [74] and the other as “reminder messaging” [75] (Table 5).

The meta-analyses results for before-after studies indicated a statistically significant RR of 1.70 (*P*<.001). Subgroup analyses, as detailed in Multimedia Appendix 5, revealed that the most efficacious intervention reported was the “reminder active call” intervention (RR 2.19; *P*<.001), followed by “educational” (RR 2.16; *P*<.001) and “multicomponent” (RR 1.61; *P*<.001) interventions. Heterogeneity and significance tests for the type of intervention are provided in Multimedia Appendices 6-8.

Meta-regression analyses were conducted for before-after studies, incorporating the following variables: quality score, publication year, continent, GNI, age category, intervention type, and follow-up time since the intervention. The variable associated with a statistically significant increase in vaccination coverage was the age category “adult-middle age” (coefficient 1.27; *P*=.01; Table 6).

**Table 4:** Characteristics of before-after included studies.^a^

Publication	Country	Recruitment/study year range	Follow-up time since intervention	Outcome (vaccination coverage)	Type of intervention	Age range	Number of patients enrolled	Quality score assigned
Gargano et al [66]	United States	2008-2009	12 months	Influenza (2008-2009 and 2009-2010)	Education (a school-based educational intervention in rural Georgia)	12-18 years	3916	8
Chen et al [76]	China	2006-2007	<6 months	HBV^b^	Education (a pilot program for HBV education in rural China)	5-12 years	2833	6
Suryadevara et al [67]	United States	2011-2012	9 months	Full series completion for all vaccines	Education (an educational intervention for resource-poor families)	0-18 years	1531	8
Toleman et al [77]	United Kingdom	2012-2014	24 months	Influenza and pneumococcal (ill patients with cancer)	Education (implementation of clinical guidelines to educate health care workers; outcome for vaccination rates in chemotherapy patients)	Adults >80 years	200	6
Sengupta et al [78]	India	2013-2014	14 months	dTPa^c^, OPV^d^, and HBV full series completion	Education (an educational intervention on the migrant population)	9-12 months	647	5
Costantino et al [79]	Italy	October 2016 to November 2016	6 months	Influenza (health care workers)	Education (an educational intervention on influenza vaccination conducted at “Paolo Giaccone” University Hospital of Palermo for the 2016/2017 seasonal influenza vaccination campaign)	18-65 years	125	7
Wallace-Brodeur et al [68]	United States	2016	36 months	HPV full series completion	Education (quality improvement and educational training of participants)	13-17 years	26,763	8
Glanternik et al [80]	United States	May 2015 to July 2015	7 months	All vaccines	Education (a training intervention of physicians to help improve communication and provide education to vaccine-hesitant parents; evaluation of outcomes for infants vaccinated)	Physicians: 24-65 years; infants: 0-6 months	13,425	7
Costantino et al [69]	Italy	October 2019 to October 2020	13 months	Influenza, dTPa, and influenza + dTPa	Education (an educational intervention during childbirth classes)	18-40 years (pregnant women)	326	8
Paunio et al [81]	Finland	1982	75 months	MMR^e^	Multicomponent (mass media and individual approach)	14 months to 6 years	562,932	6
Abd Elaziz et al [82]	Egypt	2008	1 month	MMR	Multicomponent (posters, flyers, and messages)	16-23 years (medical and nonmedical students)	651	6
Llupià et al [83]	Spain	2008-2009 influenza season	6 months	Influenza (health care workers)	Multicomponent (messages sent by emails, rewards, and a web page)	>18 years	9632	7
Cushon et al [70]	Canada	2007-2009	10 months	MMR	Multicomponent (phone calls, letters, reminders, and home visits)	14-20 months	24,540	8
Aspesi et al [84]	United States	2010-2011	9 months	Pneumococcal	Multicomponent (checklist, educational pocket cards, and handout)	All ages (hospitalized patients)	2258	6
Hu et al [85]	China	2011-2014	32 months	All vaccines	Multicomponent (a training program for vaccinators, a screening tool to identify vaccination demands among migrant clinic attendants, and social mobilization for immunization)	1-4 years (infants: n=1288; preschool: n=260)	1548	6
Baker et al [86]	United States	2013-2014	12 months	PCV^f^ 13 + PPV23^g^	Multicomponent (system-level intervention at an academic rheumatology clinic that included electronic reminders with linked order sets, physician auditing and feedback, and patient outreach)	Mean age: 57 years (patients with rheumatoid arthritis)	1255	6
Mazzoni et al [87]	United States	2010-2014	24 months	dTPa, influenza, and HPV^h^ 9 (perinatal period)	Multicomponent (stocking of immunizations in clinics, revision and expansion of standing orders, creation of a reminder/recall program, identification of an immunization champion to give direct provider feedback, expansion of a payment assistance program, and staff education)	dTPa: 20.7-33.4 years (mean age 27.4 years); influenza: 19.1-40.8 years (mean age 29.9 years); HPV 9: 19-25.1 years (mean age 22.3 years)	dTPa: 2710; influenza: 19,409; and HPV: 12,443	5
Mustafa et al [88]	Qatar	2014-2015	4 months	Influenza (health care workers and hospital B)	Multicomponent (promotional, educational, and vaccine delivery interventions; a dedicated influenza vaccination team; telephone hotline; free influenza vaccination with improved access; leadership involvement; incentives; group educational sessions; and reporting/tracking activities)	18-65 years	Hospital A: 15,341; hospital B: 16,357	6
Nzioki et al [89]	Kenya	2012-2014	18 months	All vaccines	Multicomponent (community mobilization and identification and training of volunteer community health workers; enumeration, mapping of households, and creating community units; and recruitment and training of community health extension workers)	0-1 year	833	5
Varman et al [90]	United States	2015-2016	8 months	HPV 9 full series completion	Multicomponent (clinic discussion and introduction of a multilevel intervention aiming at avoiding missed opportunities, reminder emails, and educating patients)	13-17 years (intervention on parents)	3393	6
Poscia et al [91]	Italy	2015	8 months	All vaccines	Multicomponent (a 90-minute health promotion intervention, which includes a theoretical introduction and an interactive role-play technique. Parents provided informed consent and received an invitation to a meeting with the project team)	11.3-13.3 years (mean age 12.3 years)	801	7
Kaufman et al [71]	Australia	October 2018 to December 2018	3 months	Influenza and pertussis	Multicomponent (the intervention targets all 3 levels of the health care encounter—the practice, provider, and parent levels (P3). The intervention included midwife prompts and vaccine communication training, a website, fact sheets, and parent SMS text message reminders)	21-40 years (mean age 32 years); outcome for infants	62	8
Podraza et al [92]	United States	July 2020	5 months	Meningococcal ACWY and meningococcal B	Multicomponent (multicomponent intervention)	16-19 years	335	7
Akwataghibe et al [93]	Nigeria	May 2016 to December 2016	4 months	All vaccines	Multicomponent (cycles of dialog and action between community members, frontline health workers, and local government officials in 2 wards of Remo North, facilitated by the research team)	>9 months (more than half of the child sample was aged >2 years)	340	7
Perkins et al [94]	United States	January 2017 to December 2017	12 months	HPV 9 full series completion	Multicomponent (multilevel intervention: provider training and ≥1 other evidence-based systems improvement)	13 years	3283	7
Cecinati et al [95]	Italy	2006-2007	<6 months	Influenza (ill patients with cancer)	Remind active call (telephone recall system managed by pediatricians who usually follow-up ill children with cancer)	10 years (intervention on parents)	205	7
Lam et al [96]	United States	2010-2011	1 month	dTPa	Clinical reminder (face-to-face reminder at the gynecological visit)	Adults aged >80 years and women (child-bearing age or with frequent exposure to children)	2309	7
Gattis et al [72]	United States	2011-2016	36 months	Influenza (transplanted patients)	Clinical reminder (face-to-face reminder for transplanted patients with the implementation of the transplant pharmacy vaccine program)	10.8-11.3 years	3044	8
Gossec et al [73]	France	May 2014 to October 2015	36 months	Influenza and pneumococcal	Clinical reminder (nurse visit for comorbidity counseling and for vaccination execution)	Patients with rheumatoid arthritis: 18-80 years (mean age 58.0 years)	970	8
Hernández-García and Aibar-Remón [74]	Spain	November 2014 to June 2018	—^i^	PCV 13 + PPV23	Clinical reminder (hospital vaccine consultation)	Adults aged >80 years (patients with chronic kidney disease)	101	4
Nguyen et al [75]	Vietnam	2013-2015	12 months	Timely vaccination for all vaccines	Remind messaging (SMS text message reminders)	Children	7371	4
Esteban-Vasallo et al [97]	Spain	2016 (influenza vaccination campaign)	.4 months	Influenza (patients with rare disease)	Remind messaging (SMS text message reminders)	47-68 years	106,987	7
Fairbrother et al [98]	United States	1993-1996	—	dTPa, OPV, HBV, Hib^j^, and MMR	Reward (distribution of free vaccines to health care providers)	3 months to 3 years	3211	7

^a^Variables reported were author’s first name, publication year, country, recruitment study year range, follow-up period since the intervention, outcome(s), intervention type and category, mean age range among controls/interventions, and number of enrolled patients.

^b^HBV: hepatitis B virus.

^c^dTPa: diphtheria, tetanus, and acellular pertussis.

^d^OPV: oral polio vaccine.

^e^MMR: measles, mumps, and rubella.

^f^PCV: pneumococcal conjugate vaccine.

^g^PPV: pneumococcal polysaccharide vaccine.

^h^HPV: human papillomavirus.

^i^Not available.

^j^Hib: *Hemophilus influenzae* type b.

**Table 5:** The scoring system^a^ used for before-after studies.

Scoring system	1^b^	2^c^	3^d^	4^e^	5^f^	6^g^	7^h^	8^i^	9^j^	10^k^	11^l^	Total, n
Paunio et al [81]	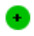	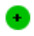	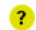	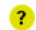	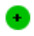	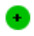	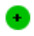	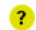	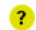	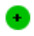	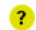	6
Fairbrother et al [98]	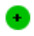	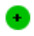	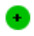	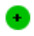	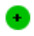	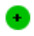	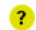	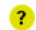	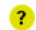	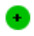	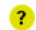	7
Abd Elaziz et al [82]	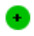	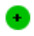	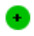	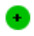	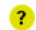	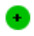	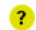	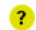	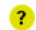	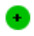	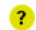	6
Llupià et al [83]	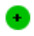	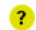	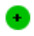	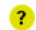	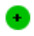	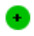	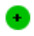	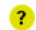	N/A^m^	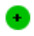	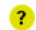	7
Cecinati et al [95]	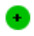	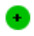	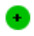	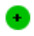	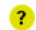	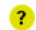	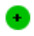	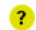	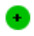	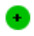	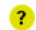	7
Gargano et al [66]	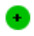	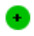	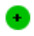	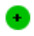	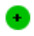	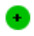	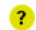	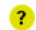	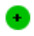	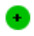	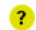	8
Chen et al [76]	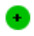	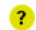	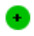	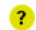	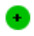	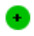	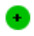	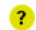	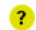	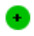	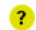	6
Cushon et al [70]	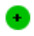	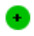	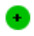	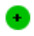	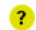	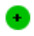	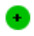	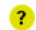	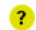	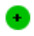	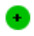	8
Lam et al [96]	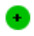	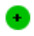	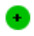	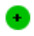	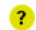	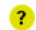	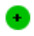	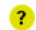	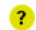	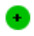	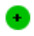	7
Suryadevara et al [67]	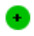	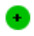	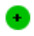	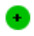	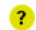	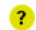	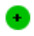	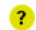	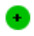	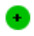	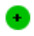	8
Aspesi et al [84]	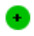	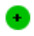	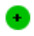	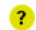	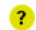	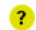	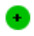	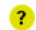	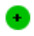	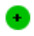	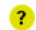	6
Toleman et al [77]	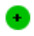	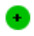	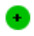	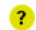	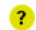	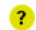	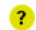	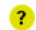	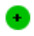	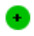	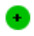	6
Hu et al [85]	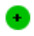	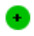	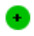	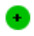	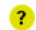	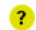	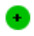	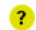	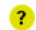	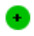	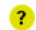	6
Baker et al [86]	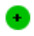	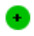	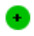	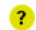	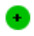	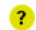	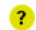	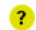	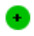	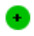	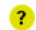	6
Mazzoni et al [87]	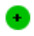	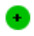	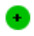	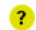	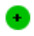	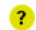	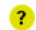	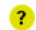	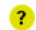	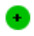	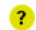	5
Nzioki et al [89]	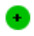	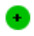	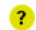	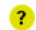	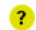	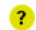	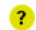	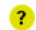	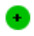	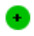	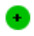	5
Nguyen et al [75]	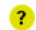	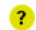	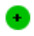	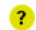	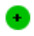	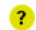	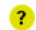	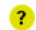	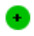	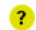	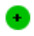	4
Mustafa et al [88]	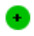	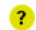	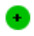	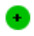	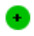	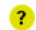	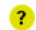	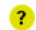	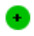	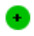	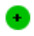	6
Sengupta et al [78]	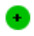	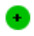	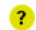	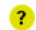	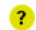	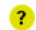	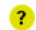	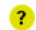	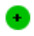	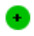	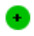	5
Varman et al [90]	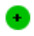	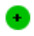	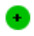	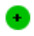	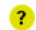	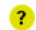	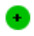	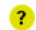	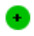	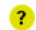	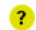	6
Gossec et al [73]	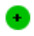	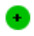	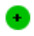	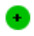	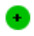	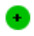	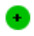	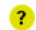	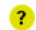	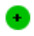	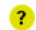	8
Esteban-Vasallo et al [97]	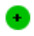	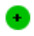	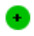	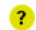	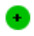	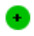	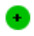	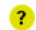	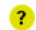	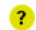	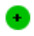	7
Poscia et al [91]	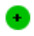	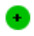	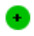	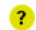	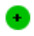	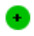	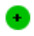	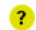	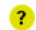	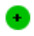	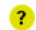	7
Costantino et al [79]	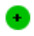	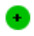	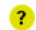	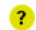	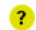	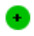	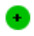	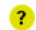	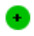	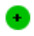	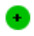	7
Gattis et al [72]	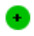	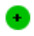	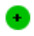	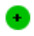	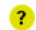	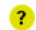	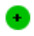	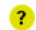	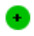	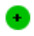	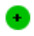	8
Wallace-Brodeur et al [68]	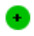	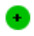	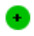	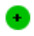	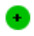	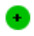	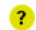	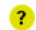	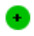	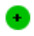	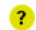	8
Glanternik et al [80]	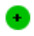	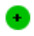	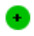	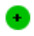	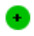	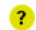	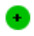	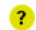	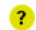	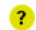	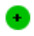	7
Kaufman et al [71]	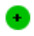	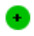	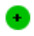	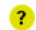	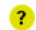	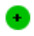	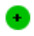	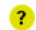	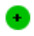	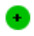	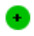	8
Costantino et al [69]	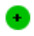	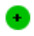	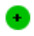	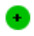	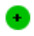	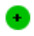	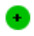	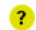	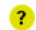	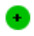	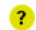	8
Podraza et al [92]	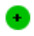	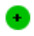	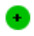	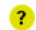	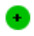	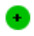	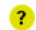	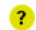	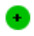	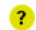	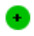	7
Akwataghibe et al [93]	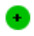	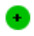	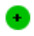	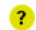	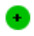	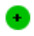	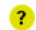	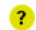	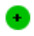	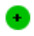	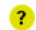	7
Perkins et al [94]	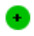	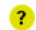	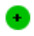	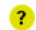	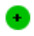	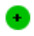	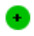	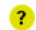	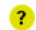	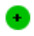	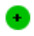	7
Hernández-García and Aibar-Remón [74]	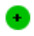	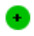	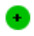	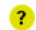	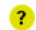	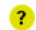	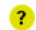	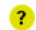	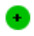	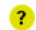	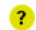	4

^a^1 additional point for the appropriate item, 0 points if inappropriate. A negative score is not expected.

^b^Was the study question or objective clearly stated?

^c^Were eligibility/selection criteria for the study population prespecified and clearly described?

^d^Were the participants in the study representative of those who would be eligible for the test/service/intervention in the general or clinical population of interest?

^e^Were all eligible participants that met the prespecified entry criteria enrolled?

^f^Was the sample size sufficiently large to provide confidence in the findings?

^g^Was the test/service/intervention clearly described and delivered consistently across the study population?

^h^Were the outcome measures prespecified, clearly defined, valid, reliable, and assessed consistently across all study participants?

^i^Were the people assessing the outcomes blinded to the participants’ exposures/interventions?

^j^Was the loss to follow-up after baseline 20% or less? Were those lost to follow-up accounted for in the analysis?

^k^Did the statistical methods examine changes in outcome measures from before to after the intervention? Were statistical tests done that provided *P* values for the pre-to-post changes?

^l^Were outcome measures of interest taken multiple times before the intervention and multiple times after the intervention (ie, did they use an interrupted time-series design)?

^m^N/A: not applicable.

**Table 6:** Meta-regression of before-after studies.

Variable				Coefficient		SE		*t* test (*df*)		*P* value		95% CI
Quality score				0.21		0.18		1.20 (45)		.24		–0.15 to 0.57
Publication year				–0.002		0.03		–0.08 (45)		.94		–0.06 to 0.05
**Continent**												
	Africa			–0.06		0.66		–0.09 (45)		.93		–1.43 to 1.31
	America			–0.14		0.33		–0.44 (45)		.67		–0.82 to 0.53
	Australia			–0.32		0.64		–0.51 (45)		.62		–0.98 to 1.63
	Asia			–0.08		0.54		–0.14 (45)		.89		–1.20 to 1.04
**Gross national income**												
	Low income			0.06		0.36		0.17 (45)		.87		–0.68 to 0.80
	Low-middle income			0.51		0.56		0.92 (45)		.37		–0.63 to 1.66
	Upper-high income			0.51		0.73		0.70 (45)		.49		–0.99 to 2.02
**Intervention type**												
	Education			–0.16		0.75		–0.21 (45)		.84		–1.70 to 1.39
	Multicomponent			–0.38		0.70		–0.54 (45)		.60		–1.84 to 1.08
	Active call			0.25		0.98		0.25 (45)		.80		–1.77 to 2.26
	Clinical remind			–0.67		0.84		–0.80 (45)		.43		–2.40 to 1.05
	Remind messaging			–0.45		0.86		–0.52 (45)		.61		–2.23 to 1.33
**Age**												
	Infant-preschool			–0.19		0.31		–0.61 (45)		.55		–0.83 to 0.45
	Children-adolescent			–0.05		0.29		–0.17 (45)		.87		–0.65 to 0.55
	Adult-middle age			1.27		0.28		4.54 (45)		<.001^a^		0.70 to 1.84
	Aged			–0.75		0.38		–1.95 (45)		.06		–1.54 to 0.04
**Follow-up**												
		12 months	0.37		0.32		1.17 (45)		.25		–0.29 to 1.03	
		>12 months	0.05		0.32		0.14 (45)		.89		–0.62 to 0.71	

^a^Statistically significant results.

## Discussion

### Principal Findings

Catch-up vaccination strategies are crucial components of a comprehensive national immunization program and should be continually integrated [99,100]. Understanding the effectiveness of vaccination interventions is essential for selecting those best suited to diverse sociodemographic contexts. Therefore, this research included RCTs and before-after studies, recognized as effective catch-up strategies.

In general, catch-up interventions identified in the studies, categorized into 4 groups (“multicomponent,” “educational,” “remind,” and “reward studies”), demonstrated effectiveness in promoting adherence to vaccination. However, practices associated with certain types of “reward” interventions did not exhibit statistical significance [37,38]. Reminder and recall interventions are used to prompt individuals within a target population regarding upcoming vaccinations (recall) or overdue vaccinations (reminder). These strategies vary in content based on the type of vaccination and the target demographic. They are implemented through various methods, including telephone calls with active reminders; messaging via SMS text messages, emails, or traditional mails; in-person reminders within clinical settings; the use of physical objects as reminders; and reminders via web-based platforms. Active call interventions have been shown to significantly enhance vaccination adherence rates. This is likely because telephone contact provides direct access to health care professionals who can address patients’ concerns and inquiries. Moreover, active calls serve as a reminder tool that is easily accessible and adaptable, even in resource-constrained settings. For instance, a study conducted in Nigeria demonstrated that vaccination uptake doubled among newborns whose mothers received calls from health care workers at vaccination centers [34].

Vaccination reminder interventions conducted through messaging (SMS text messages, emails, letters, and notifications) are also effective, although they tend to have a lesser impact compared with “active calls” [36]. This could be attributed to the limitations of “messaging reminders” interventions, such as the inability to engage in immediate question-and-answer discussions with health care workers and the challenge of personalizing the SMS text message, which is often predefined and sent using automated software [41]. Despite the limitations, the widespread use of mobile technologies enables rapid and effortless communication with large communities of people. Additionally, considering the low cost and ubiquity of mobile phones, “messaging reminders” could prove to be an excellent strategy for implementation, especially in low- and middle-income countries [54,63].

Another highly effective strategy for increasing vaccination coverage is the “clinical-remind” approach, where all vaccination promotion interventions occur directly within hospital or primary care settings. Offering vaccination to patients during hospital visits for examinations, consultations, or treatments is a strategy endorsed by the World Health Organization (WHO) to minimize missed opportunities for vaccination. This approach aims to enhance health care service delivery and foster seamless collaboration among health care professionals [101]. A randomized study conducted in Georgia in 2018 serves as a demonstration of this strategy. In the study, the tetanus, diphtheria, and pertussis (Tdap) vaccine was offered to pregnant women during gynecological visits in antenatal clinics. Results indicated a higher willingness to receive vaccination among pregnant women in the intervention group compared with the control arm [56]. Presently, the Advisory Committee on Immunization Practices (ACIP) recommends that pregnant women receive the Tdap vaccine during each pregnancy, regardless of their immunization history [102]. Despite recommendations, maternal Tdap vaccination coverage remains low, not only in the United States but also in many other parts of the world. However, evidence suggests that vaccination strategies involving patient engagement in a clinical setting can effectively increase coverage [56].

The results further highlight the effectiveness of “educational interventions” in vaccine catch-up efforts. Health education stands as one of the primary tools for ensuring that a population has access to health care services. As early as 1983, the WHO recognized health education as a universal right of communities [103]. This right can be realized through integrated information and education programs, aiming to enhance both the population’s desire for good health and their ability to discern the validity of the information they receive [104]. Lack of knowledge and misinformation stand as the primary barriers impeding widespread access to vaccination [12]. Addressing these challenges necessitates a shift toward a more suitable educational approach within the vaccination context. Particularly, among the selected educational interventions, those based on face-to-face dialog between patients and health care professionals emerge as the most effective. An RCT conducted in Italy, aimed at evaluating the impact of various types of educational programs targeting the adolescent population, demonstrated that face-to-face lessons are more effective in increasing vaccination coverage compared with web-based lessons [45].

A highly effective strategy for boosting vaccination coverage is the multidimensional approach, which emerged as the most frequently utilized in the studies included in this review. This approach encompasses interventions that combine vaccination reminder tools with awareness sessions and patient education on vaccination. These interventions are implemented through multiple steps and in various formats. According to the Strategic Advisory Group of Experts on Immunization (SAGE), “multicomponent” interventions are more effective than those with a single component. By addressing various aspects, they are more successful in enhancing knowledge and awareness and in fostering psychological shifts and attitude changes toward vaccinations [104]. A compelling example comes from a before-after study conducted at a rheumatology clinic in Illinois, United States. This study implemented a multifaceted intervention, which included electronic reminders with linked order sets, physician auditing, and patient outreach, resulting in improved patient vaccination rates [86].

The meta-regression analysis indicated a higher effectiveness of vaccination interventions in the European continent compared with other geographic regions. This finding is likely a reflection of the disparity in economic resources and access to health care services between high-income and low-middle-income countries [4]. There is no one-size-fits-all approach to vaccine catch-up that proves effective across all contexts and realities. This is especially true for developing countries, where scientific evidence remains limited. Many vaccine interventions in the WHO African Region, reviewed by the SAGE to develop guidelines addressing vaccine hesitancy, are often documented in the gray literature, which was not encompassed in our review [104]. However, this research has unveiled findings that hold potential global relevance. For instance, health strategies centered around reminders have proven to be effective and cost-efficient, making them particularly suitable for countries with limited resources [54]. Conversely, the significant success observed in certain vaccination programs conducted in the European continent underscores the importance of evaluating the performance of vaccination services in these countries [45,79,83,97]. As of now, there remains a lack of standardized protocols for immunization services aimed at monitoring and improving service quality. Progress in this area has stagnated since the drafting of the National Vaccine Advisory Committee’s standards for vaccination services in the 1980s [105]. However, some authors have proposed models to develop accreditation manuals for vaccination services. These manuals would establish a minimum set of quality standards to ensure the delivery of high-quality preventive health care services. Such standards are crucial for optimizing service effectiveness and ensuring the efficient allocation of economic resources toward public health initiatives [106,107].

Finally, the meta-regression results also revealed the effectiveness of various interventions aimed at vaccination catch-up among a specific demographic: middle-aged adults. This finding may be attributed to the organization of vaccination services by age group. Historically, the majority of vaccines have been developed for pediatric populations, and extensive national and international collaborative efforts have strongly supported mass immunization of the youngest individuals, even in developed countries, to ensure adequate access to life-saving vaccines. Moreover, childhood vaccination programs are typically uniform, well-defined, and bolstered by annual monitoring of vaccine dissemination and its impact on reducing morbidity and mortality. Conversely, reaching the adult population with standard vaccine delivery methods can be more challenging. However, catch-up vaccination and recall interventions have the potential to be highly effective in enhancing coverage among adults.

The older age population, aged 65 years and over, faces a lack of dedicated vaccination programs tailored for their age group, despite being the demographic most vulnerable to the risks and complications associated with infectious diseases [108]. In 2009, 2 geriatric societies, the European Geriatric Medicine Society (EUGMS) and the International Association of Gerontology and Geriatrics-European Region (IAGGER), formulated guidelines outlining recommended vaccinations for the geriatric population. Despite these efforts, the vaccination provision for older patients continues to vary widely among different European countries [109]. The ongoing challenge is to develop vaccination programs for older adults that are as comprehensive and effective as those designed for children.

Moreover, numerous vaccination catch-up interventions rely on “reminder” strategies, which entail sending SMS text messages or emails and providing information through dedicated web pages [25,35,97]. While the utilization of mobile technologies can be a significant asset for vaccination strategies targeting adolescents and adults, it may pose a challenge for the older age population. Alternative intervention modalities may thus prove more suitable. For instance, a study conducted in France aimed at offering recommended vaccinations to hospitalized patients demonstrated a significant increase in vaccination coverage [57]. Providing vaccination counseling in hospital or outpatient settings could be the most effective strategy for achieving widespread vaccination among the older age population.

### Comparison With Prior Studies

We identified several prior meta-analyses, published between 2017 and 2018, that investigated the impact of vaccine interventions targeting specific population groups or focused on particular types of interventions [3]. Although patient reminder and recall systems have been extensively studied, the existing literature does not provide data on the potential impact of other vaccine catch-up interventions [110]. Moreover, the search strategy for this systematic review was not confined to any specific geographical context or timeframe, nor was it designed to target a particular type of vaccination. Our study offers an analysis of various vaccination strategies, enabling the identification of the most suitable interventions for each population and vaccination category, across diverse sociodemographic contexts.

### Limitations

The search strategy for this systematic review did not encompass gray literature documents and reports. Although the investigation covered all geographical contexts, the majority of the selected and included studies were conducted in developed countries. This bias may arise because some studies on vaccinations and recall interventions in developing countries are typically found in gray literature. Furthermore, no vaccination catch-up interventions related to the COVID-19 vaccine were included. The COVID-19 vaccination, being introduced during an emergency pandemic situation, is not part of the scheduled vaccination regimen. Despite these limitations, this study is among the first to investigate a broad array of vaccine catch-up strategies for scheduled vaccines, encompassing recent studies targeting all age groups of the population.

### Conclusions

Vaccination reminder interventions, incorporating educational sessions for the population and utilizing various reminder methods such as SMS text messages and calls, as well as multifaceted interventions combining multiple strategies, have demonstrated effectiveness in enhancing vaccination coverage. However, it is important to note that there is no universal catch-up strategy that performs well across all contexts and realities. It is essential to adopt the most suitable intervention strategy based on the patient category, resource availability, and the socioeconomic status of the target population to be vaccinated.

## Appendix

Multimedia Appendix 1 PRISMA (Preferred Reporting Items for Systematic Reviews and Meta-Analysis) checklist.

Multimedia Appendix 2 Search strategy.

Multimedia Appendix 3 Heterogeneity test for randomized controlled trial included studies.

Multimedia Appendix 4 Forest plots of intervention for randomized controlled trial included studies.

Multimedia Appendix 5 Forest plots of intervention for before-after included studies.

Multimedia Appendix 6 Funnel plot of randomized controlled trial included studies.

Multimedia Appendix 7 Heterogeneity test for before-after included studies.

Multimedia Appendix 8 Funnel plot of before-after included studies.

## Abbreviations

ACIP: Advisory Committee on Immunization Practices
EUGMS: European Geriatric Medicine Society
GNI: gross national income
GRADE: Grading of Recommendations Assessment, Development, and Evaluation
HPV: human papillomavirus
IAGGER: International Association of Gerontology and Geriatrics-European Region
MeSH: Medical Subject Headings
PICOS: Population, Intervention, Comparison, Outcomes and Study
PRISMA: Preferred Reporting Items for Systematic Reviews and Meta-Analysis
RCT: randomized controlled trial
RR: risk ratio
SAGE: Strategic Advisory Group of Experts on Immunization
Tdap: tetanus, diphtheria, and pertussis
VCIE: vaccination coverage improvement effectiveness
WHO: World Health Organization
