# Supplementary material for: Effectiveness of Catch-Up Vaccination Interventions Versus Standard or Usual Care Procedures in Increasing Adherence to Recommended Vaccinations Among Different Age Groups: Systematic Review and Meta-Analysis of Randomized Controlled Trials and Before-After Studies
Source: JMIR Public Health Surveill. 2024 Jul 23;10:e52926. doi: 10.2196/52926 (PMC11303899; doi:10.2196/52926)
Supplement: Multimedia Appendix 2 [file publichealth_v10i1e52926_app2.docx]

Multimedia Appendix 2

**Scopus database**: [“vaccination*” OR “vaccine”] AND [“effectiveness” OR “efficacy”] AND [[[“catch”] AND [“up”]] OR [[“mop”] AND [“up”]] OR [[“keep”] AND [“up”]] OR [[“coverage”] AND [“improvement”]]] AND [“intervention”]

**PubMed/MEDLINE database**: [[["vaccin"[SupplementaryM Concept] OR "vaccin" [All Fields] OR "vaccination" [MeSH Terms] OR "vaccination" [All Fields] OR "vaccinable"[All Fields] OR "vaccinal"[All Fields] OR "vaccinate" [All Fields] OR "vaccinated" [All Fields] OR "vaccinates" [All Fields] OR "vaccinating" [All Fields] OR "vaccinations"[All Fields] OR "vaccination s" [All Fields] OR "vaccinator"[All Fields] OR "vaccinators"[All Fields] OR "vaccine s"[All Fields] OR "vaccined" [All Fields] OR "vaccines" [MeSH Terms] OR "vaccines" [All Fields] OR "vaccine" [All Fields] OR "vaccins" [All Fields] OR ["vaccin" [Supplementary Concept] OR "vaccin" [All Fields] OR "vaccination" [MeSH Terms] OR "vaccination" [All Fields] OR "vaccinable" [All Fields] OR "vaccinal" [All Fields] OR "vaccinate" [All Fields] OR "vaccinated" [All Fields] OR "vaccinates" [All Fields] OR "vaccinating" [All Fields] OR "vaccinations" [All Fields] OR "vaccinations" [All Fields] OR "vaccinator" [All Fields] OR "vaccinators" [All Fields] OR "vaccines" [All Fields] OR "vaccined" [All Fields] OR "vaccines" [MeSH Terms] OR "vaccines" [All Fields] OR "vaccine" [All Fields] OR "vaccins" [All Fields]]] AND ["effect" [All Fields] OR "effecting" [All Fields] OR "effective" [All Fields] OR "effectively" [All Fields] OR "effectiveness" [All Fields] OR "effectivenesses" [All Fields] OR "effectives" [All Fields] OR "effectivities" [All Fields] OR "effectivity" [All Fields] OR "effects" [All Fields] OR ["efficacies" [All Fields] OR "efficacious" [All Fields] OR "efficaciously" [All Fields] OR "efficaciousness" [All Fields] OR "efficacy" [All Fields]]]] AND [[["catch" [All Fields] OR "catches" [All Fields] OR "catching" [All Fields]] AND "up" [All Fields]] OR ["mop" [All Fields] AND "up" [All Fields]] OR ["keep" [All Fields] AND "up" [All Fields]] OR [["coverage" [All Fields] OR "coverages" [All Fields]] AND ["improve" [All Fields] OR "improved" [All Fields] OR "improvement" [All Fields] OR "improvements" [All Fields] OR "improves" [All Fields] OR "improving" [All Fields] OR "improvment" [All Fields]]]]] AND ["intervention s"[All Fields] OR "interventions"[All Fields] OR "interventive"[All Fields] OR "methods" [MeSH Terms] OR "methods" [All Fields] OR "intervention" [All Fields] OR "interventional" [All Fields]].
