# Supplementary material for: Effectiveness of Catch-Up Vaccination Interventions Versus Standard or Usual Care Procedures in Increasing Adherence to Recommended Vaccinations Among Different Age Groups: Systematic Review and Meta-Analysis of Randomized Controlled Trials and Before-After Studies
Source: JMIR Public Health Surveill. 2024 Jul 23;10:e52926. doi: 10.2196/52926 (PMC11303899; doi:10.2196/52926)
Supplement: Multimedia Appendix 3 [file publichealth_v10i1e52926_app3.docx]

Multimedia Appendix 3

|  | *Heterogeneity tests* | | | | | *Significance tests of RR=1* | |
| --- | --- | --- | --- | --- | --- | --- | --- |
| *Intervention type* | *Statistic heterogeneity* | *Degrees of freedom* | *p* | *I-squared* |  | *Z* | *p-value* |
| *Multicomponent* | *1367.95* | *26* | *0.000* | *98.1%* |  | *5.83* | ***0.000**** |
| *Remind clinical* | *496.66* | *12* | *0.000* | *97.6%* |  | *7.13* | ***0.000**** |
| *Remind web* | *12.20* | *6* | *0.058* | *50.8%* |  | *0.44* | *0.663* |
| *Remind messaging* | *52.88* | *14* | *0.000* | *73.5%* |  | *3.04* | ***0.002**** |
| *Remind active call* | *18.49* | *1* | *0.000* | *95.2%* |  | *0.46* | *0.649* |
| *Educational* | *175.40* | *13* | *0.000* | *92.6%* |  | *6.16* | ***0.000**** |
| *Remind object* | *2.66* | *4* | *0.616* | *0.0%* |  | *1.50* | *0.133* |
| *Reward* | *10.60* | *1* | *0.001* | *90.6%* |  | *1.56* | *0.117* |
| *Overall* | *2394.10* | *84* | *0.000* | *96.5%* |  | *14.57* | ***0.000**** |

Heterogeneity and significance tests for RCT included studies; * = statistically significant results
