# Supplementary material for: Effectiveness of Catch-Up Vaccination Interventions Versus Standard or Usual Care Procedures in Increasing Adherence to Recommended Vaccinations Among Different Age Groups: Systematic Review and Meta-Analysis of Randomized Controlled Trials and Before-After Studies
Source: JMIR Public Health Surveill. 2024 Jul 23;10:e52926. doi: 10.2196/52926 (PMC11303899; doi:10.2196/52926)

Multimedia Appendix 4

**(A):** Forest plots of multicomponent intervention for RCT included studies.


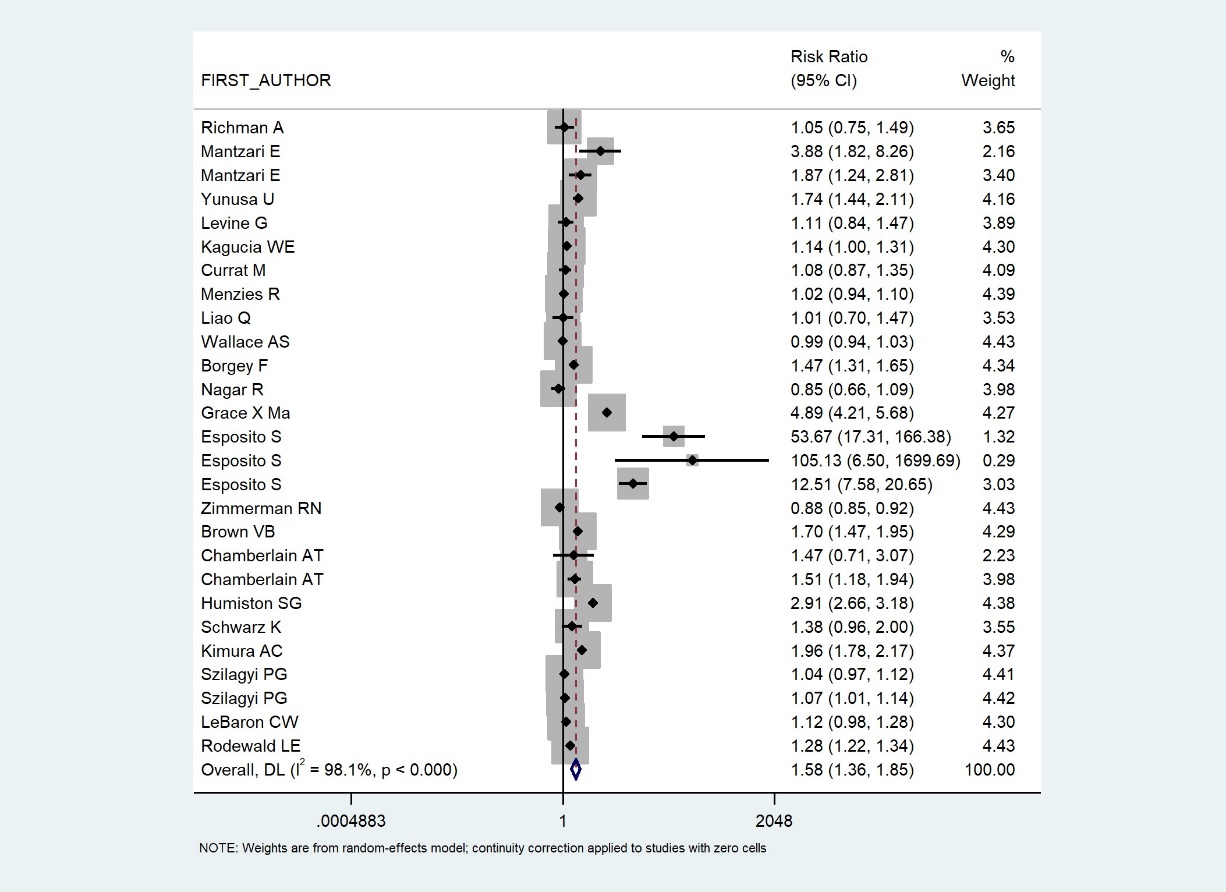


**(B)** Forest plots of remind clinical intervention for RCT included studies


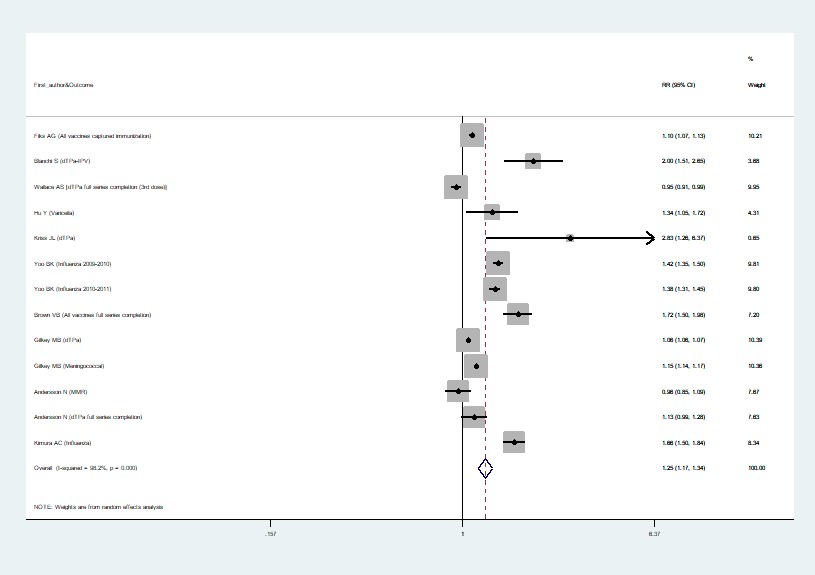


**(C)** Forest plots of educational intervention for RCT included studies


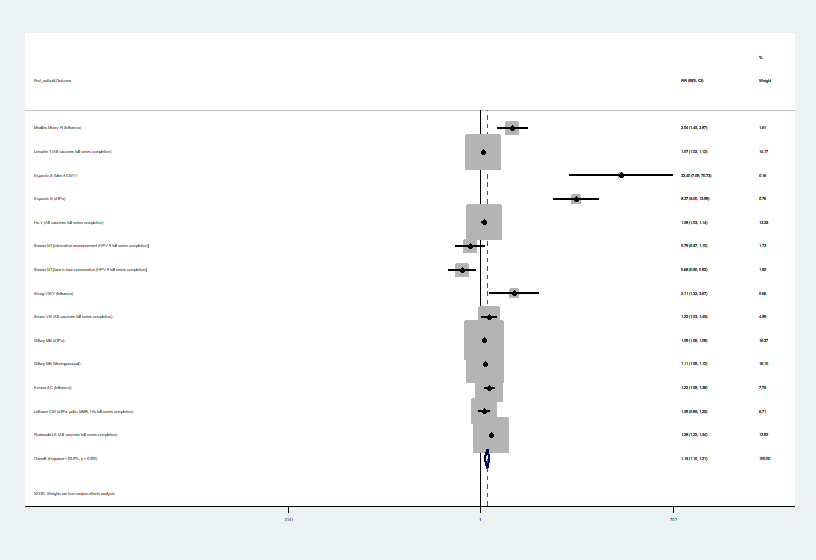


**(D)** Forest plots of messaging intervention for RCT included studies


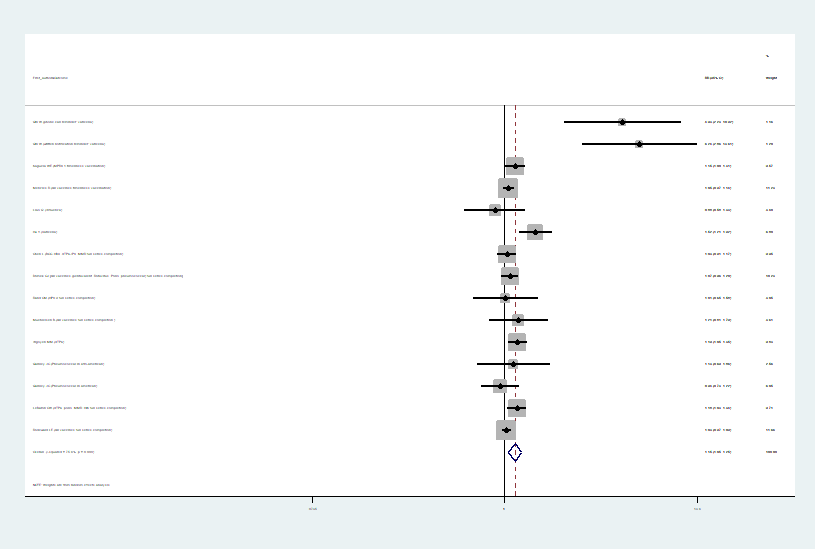

Supplement: Multimedia Appendix 4 [file publichealth_v10i1e52926_app4.docx]
