# Supplementary figures and images for: Effectiveness of Catch-Up Vaccination Interventions Versus Standard or Usual Care Procedures in Increasing Adherence to Recommended Vaccinations Among Different Age Groups: Systematic Review and Meta-Analysis of Randomized Controlled Trials and Before-After Studies
Source: JMIR Public Health Surveill. 2024 Jul 23;10:e52926. doi: 10.2196/52926 (PMC11303899; doi:10.2196/52926)

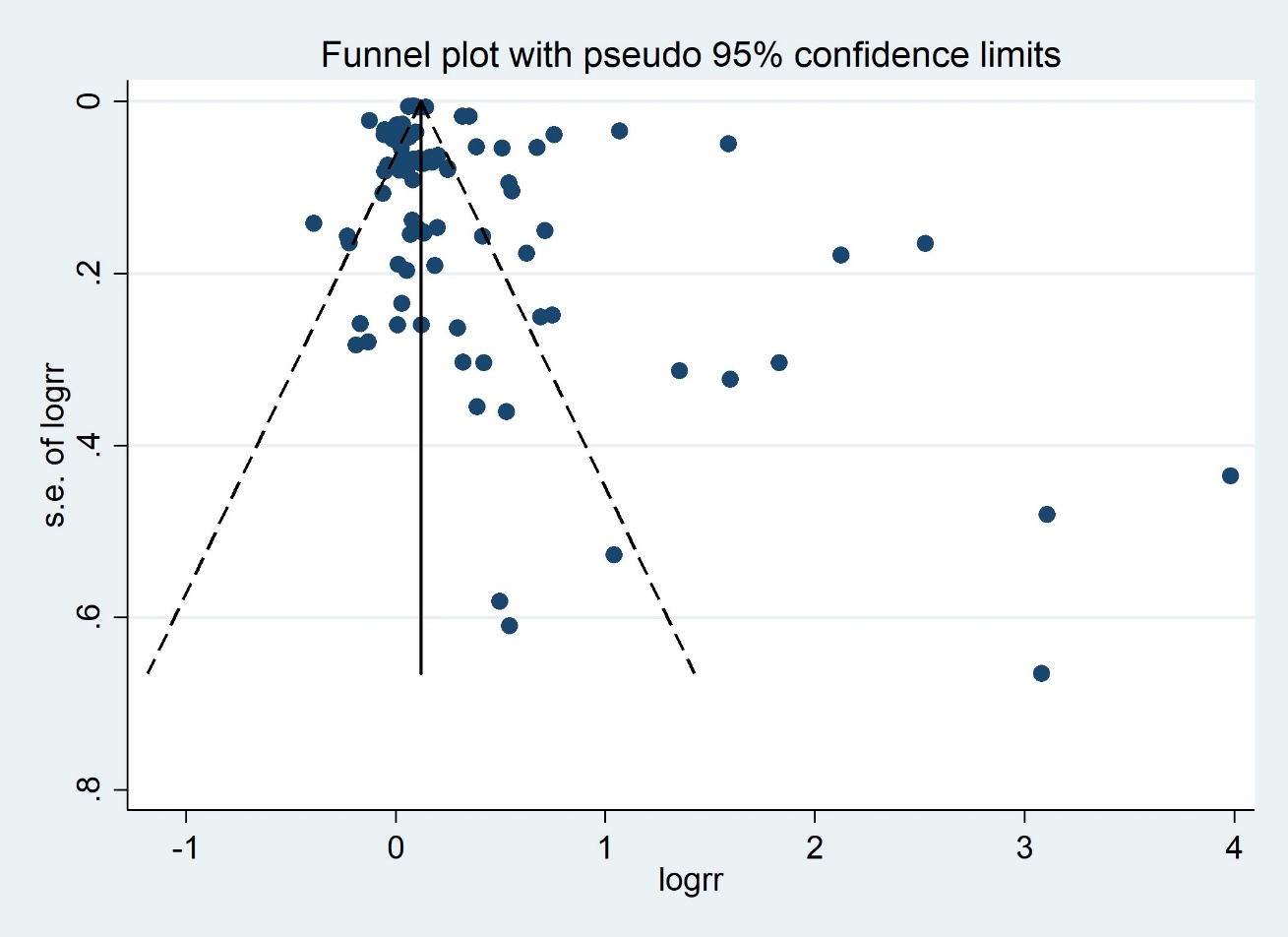

Supplement: Multimedia Appendix 6 [file publichealth_v10i1e52926_app6.docx]

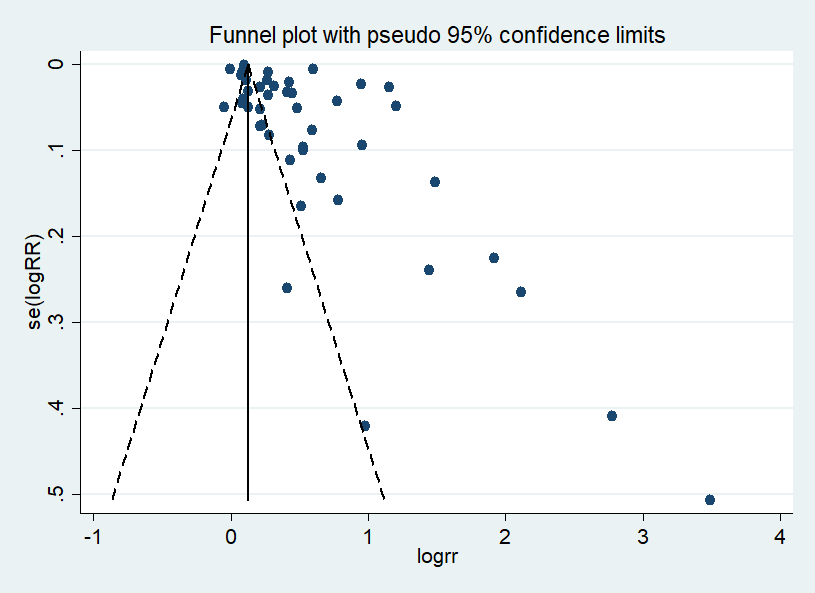

Supplement: Multimedia Appendix 8 [file publichealth_v10i1e52926_app8.docx]
