# Supplementary material for: Effectiveness of Catch-Up Vaccination Interventions Versus Standard or Usual Care Procedures in Increasing Adherence to Recommended Vaccinations Among Different Age Groups: Systematic Review and Meta-Analysis of Randomized Controlled Trials and Before-After Studies
Source: JMIR Public Health Surveill. 2024 Jul 23;10:e52926. doi: 10.2196/52926 (PMC11303899; doi:10.2196/52926)
Supplement: Multimedia Appendix 7 [file publichealth_v10i1e52926_app7.docx]

Multimedia Appendix 7

|  | *Heterogeneity tests* | | | | | *Significance tests of RR=1* | |
| --- | --- | --- | --- | --- | --- | --- | --- |
| *Intervention type* | *Statistic heterogeneity* | *Degrees of freedom* | *p* | *I-squared* |  | *Z* | *p-value* |
| *Multicomponent* | *5464.19* | *20* | *0.000* | *99.6%* |  | *7.69* | ***0.000**** |
| *Remind clinical* | *77.42* | *4* | *0.000* | *94.8%* |  | *3.07* | ***0.002**** |
| *Remind messaging* | *2639.00* | *2* | *0.000* | *99.9%* |  | *1.85* | *0.065* |
| *Remind active call* | *0.00* | *0* | *-* | *-* |  | *4.97* | ***0.000**** |
| *Educational* | *1008.63* | *13* | *0.000* | *95.7%* |  | *5.12* | ***0.000**** |
| *Reward* | *0.00* | *0* | *-* | *-* |  | *3.46* | ***0.001**** |
| *Overall* | *25995.55* | *45* | *0.000* | *99.8%* |  | *10.92* | ***0.000**** |

Heterogeneity and significance tests for before-after included studies; * = statistically significant results
